# A new technological approach in diagnostic pathology: mass spectrometry imaging-based metabolomics for biomarker detection in urachal cancer

**DOI:** 10.1038/s41374-021-00612-7

**Published:** 2021-05-21

**Authors:** Judith Martha Neumann, Karsten Niehaus, Nils Neumann, Hans Christoph Knobloch, Felix Bremmer, Ulrich Krafft, Udo Kellner, Peter Nyirády, Tibor Szarvas, Hanna Bednarz, Henning Reis

**Affiliations:** 1 Proteome and Metabolome Research, Center for Biotechnology (CeBiTec), Faculty of Biology, Bielefeld University, Bielefeld, Germany; 2 Research Institute for Cognition and Robotics (CoR-Lab), Bielefeld University, Bielefeld, Germany; 3 Department of Urology, University Medical Center Göttingen, University of Göttingen, Göttingen, Germany; 4 Institute of Pathology, University Medical Center Göttingen, University of Göttingen, Göttingen, Germany; 5 Department of Urology, University Hospital Essen, University of Duisburg-Essen, Essen, Germany; 6 Institut für Pathologie, Johannes Wesling Klinikum Minden, Minden, Germany; 7 Department of Urology, Semmelweis University Budapest, Budapest, Hungary; 8 Medical School OWL, Bielefeld University, Bielefeld, Germany; 9 Institute of Pathology, University Hospital Essen, University of Duisburg-Essen, Essen, Germany

**Keywords:** Cancer metabolism, Tumour biomarkers, Urological cancer

## Abstract

Urachal adenocarcinomas (UrC) are rare but aggressive. Despite being of profound therapeutic relevance, UrC cannot be differentiated by histomorphology alone from other adenocarcinomas of differential diagnostic importance. As no reliable tissue-based diagnostic biomarkers are available, we aimed to detect such by integrating mass-spectrometry imaging-based metabolomics and digital pathology, thus allowing for a multimodal approach on the basis of spatial information. To achieve this, a cohort of UrC (*n* = 19) and colorectal adenocarcinomas (CRC, *n* = 27) as the differential diagnosis of highest therapeutic relevance was created, tissue micro-arrays (TMAs) were constructed, and pathological data was recorded. Hematoxylin and eosin (H&E) stained tissue sections were scanned and annotated, enabling an automized discrimination of tumor and non-tumor areas after training of an adequate algorithm. Spectral information within tumor regions, obtained via matrix-assisted laser desorption/ionization (MALDI)-Orbitrap-mass spectrometry imaging (MSI), were subsequently extracted in an automated workflow. On this basis, metabolic differences between UrC and CRC were revealed using machine learning algorithms. As a result, the study demonstrated the feasibility of MALDI-MSI for the evaluation of FFPE tissue in UrC and CRC with the potential to combine spatial metabolomics data with annotated histopathological data from digitalized H&E slides. The detected Area under the curve (AUC) of 0.94 in general and 0.77 for the analyte taurine alone (diagnostic accuracy for taurine: 74%) makes the technology a promising tool in this differential diagnostic dilemma situation. Although the data has to be considered as a proof-of-concept study, it presents a new adoption of this technology that has not been used in this scenario in which reliable diagnostic biomarkers (such as immunohistochemical markers) are currently not available.

## Abstract (web summary)

New diagnostic strategies are needed to distinguish rarely arising urachal adenocarcinomas (UrC) from other types of adenocarcinomas, as therapy regimes differ. In situ metabolic differences between UrC and colorectal adenocarcinomas were revealed using mass spectrometry imaging combined with digital pathology. A classification accuracy of 87% was achieved, exceeding currently available approaches.

## Introduction

The urachus is a fetal structure that connects the forming urinary bladder to the allantois during early intra-uterine development. It obliterates to form the median umbilical ligament and runs from the roof of the bladder to the umbilicus in the midline within the space of Retzius [1]. While macroscopic residues are uncommon, microscopic urachal remnants can be detected in up to 32% of adults [2]. With an incidence of <1 case per 1,000,000 people per year, urachal cancer can rarely arise from these remnants with urachal adenocarcinomas (UrC) accounting for over 90% of cases [3–6]. Non-cystic type UrC mostly (57%) exhibit a mucinous histology followed by intestinal, not otherwise specified (NOS), mixed, and signet ring cell histology subtypes [4, 7]. These histological subtypes, however, show striking overlaps with other types of adenocarcinomas. This can pose a major differential diagnostic problem in the histopathological evaluation of biopsies from this region. However, the correct distinction from other tumors is vital as the therapy regimes differ. For example, and most importantly, a colorectal adenocarcinoma (CRC) growing into the bladder mostly represents a palliative situation while localized UrC can be cured by partial cystectomy with resection of the median umbilical ligament and umbilicus. As in this specific setting immunohistochemistry is of little utility and radiology and clinical examination often are non-conclusive, tissue based diagnostic biomarkers are urgently needed to allow a correct pre-operative diagnosis and individual therapy planning [4]. We therefore sought to identify metabolic diagnostic biomarkers using mass spectrometry imaging (MSI), which has not been performed in this field.

The tumor metabolism is known to differ from metabolism of corresponding normal cells [8]. Reprogramming of the energy metabolism is one of the hallmarks of cancer [9], including elevated glutaminolysis [10] and enhanced glycolysis rates even under aerobic conditions, known as the Warburg effect [11]. The altered metabolism of cancer is necessary for the enhanced proliferation rate of tumor cells [12, 13]. To analyze metabolic alterations in situ, matrix-assisted laser desorption/ionization (MALDI) MSI is a powerful tool [14]. Depending on the applied matrix, different types of analytes can be detected on a single tissue section. By scanning the section with a laser and combining with the pixel-wise sensitive detection by mass spectrometry, the spatial information of various metabolites in the tissue is revealed. The combination of data obtained from MSI with histopathological information, known as multimodal imaging, is crucial to avoid artefacts in data analysis [15] and allows for correct classification of profiles in cancer and non-cancerous tissue. Highly detailed histological or immunohistochemical data for co-registrations can be obtained by digital pathology accompanied by significant annotations by pathologists [16].

Multimodal analysis of MSI data with fluorescent image data from a digital pathology software was demonstrated lately [17]. A similar approach was additionally recently described for the combination of MSI data with digital pathology information from hematoxylin and eosin (H&E) stained slides [18]. This is relevant as H&E-staining of tissue specimen represents a routine histological technique of high informative value on cellular and non-cellular level, in context of tissue structure and composition. We therefore, for the first time, used this new technological approach to detect diagnostic biomarkers in a critical tissue based differential diagnostic setting, focusing on the discrimination of UrC and CRC.

## Material and methods

### Cohort and construction of tissue micro-arrays

A cohort of UrC and CRC was retrospectively collected from the archive of the Institute of Pathology at the University Hospital Essen (UrC: *n* = 14, CRC: *n* = 27) and of the Institute of Pathology at the University Hospital Göttingen (UrC: *n* = 5). Details on clinico-pathological data are given in Table 1. Diagnoses of UrC and CRC were established following WHO criteria [19, 20]. Histopathological information was compiled after review by a genitourinary (GU) pathologist (HR). Tumor areas were marked on the H&E slides (HR) and TMAs were constructed using an automated platform (TMA Grand Master, 3DHISTECH, Budapest, Hungary) with three cores per case (diameter: 1.3 mm). The study was approved by the ethics committee of the University of Duisburg-Essen (15-6372-BO) and it was performed in accordance with the Helsinki declaration and its amendments.

**Table 1:** Clinico-pathological data of the cohort with urachal adenocarcinomas (UrC) and colorectal adenocarcinomas (CRC).

		UrC	CRC
		*N* (%)	*N* (%)
Sex	Female	8 (42)	11 (41)
	Male	11 (58)	16 (59)
	Total	19	27
Age (median, y)		50	74
Sheldon stage	I	0 (0)	
	II	0 (0)	
	IIIA	7 (37)	
	IIIB	0 (0)	
	IIIC	1 (5)	
	IIID	0 (0)	
	IVA	1 (5)	
	IVB	6 (32)	
	n/a	4 (21)	
	Total	19	
Mayo stage	I	3 (16)	
	II	5 (26)	
	III	1 (5)	
	IV	6 (32)	
	n/a	4 (21)	
	Total	19	
TNM tumor stage	pT1		0 (0)
	pT2		5 (19)
	pT3		15 (56)
	pT4		7 (26)
	Total		27
TNM lymph node status	pN0		14 (52)
	pN1		5 (19)
	pN2		8 (30)
	Total		27
Grade	G1	1 (5)	3 (11)
	G2	14 (74)	18 (67)
	G3	4 (21)	6 (22)
	Total	19	27
Subtype	Intestinal	5 (26)	25 (93)
	Mucinous	7 (37)	2 (7)
	Signet ring cell	2 (11)	0 (0)
	NOS	4 (21)	0 (0)
	Mixed	1 (5)	0 (0)
	Total	19	27

Tumor stage of UrC is indicated using the Sheldon [39] or Mayo staging system [40] as commonly accepted. The Tumor, Node, Metastasis (TNM) system in its 7th edition was used for staging of CRC [41].

*y* years, *n/a* data not available, *NOS* not otherwise specified.

### Digital pathology

TMAs were sectioned and H&E stained on a HE600 platform (Ventana/Roche diagnostics, Oro valley, AZ, USA) using standard diagnostic protocols. Stained TMAs were scanned using an Aperio AT2 system (Leica Biosystems, Wetzlar, Germany) for creation of digital whole slide images (WSIs). WSIs were annotated by a GU-pathologist (HR) using the software QuPath v0.1.2 [21] as basis of adjustment of automated tumor detection thresholds (JMN). After TMA dearraying and cell detection, smoothed features were calculated for 25 µm and 50 µm. The classifier was trained on tumor and non-tumor regions. The random trees classifier was built with 23,755 training objects and classification results were verified by the pathologist.

### Mass spectrometry imaging

Serial TMA sections (4 µm thickness) were cut onto indium tin oxide coated glass slides (Bruker Daltonik GmbH, Bremen, Germany) using fresh blades for every new block. Sections were stored at 4 °C in the dark until further use. Directly before matrix application, TMA sections were deparaffinized twice for 8 min in reagent grade xylene, as described by others [22]. Matrix N-(1-naphthyl) ethylenediamine dihydrochloride (NEDC) (≥99% p. a., Carl Roth GmbH + Co. KG, Karlsruhe, Germany) was used at a concentration of 7 mg/ml in Methanol/Water (70/30, v/v). Matrix application was executed using the TM-Sprayer (HTX Technologies, LLC, Chapel Hill, USA) with a flow rate of 0.12 ml/min, a velocity of 1200 mm/min, and 3 mm track spacing for 30 passes at a nozzle temperature of 70 °C. Samples were stored in a dry cabinet (Eureka Dry Tech/Taiwan Dry Tech, Taipei City, Taiwan) until measurements.

MALDI-Orbitrap-MSI was performed on a Spectroglyph MALDI/ESI Injector (Spectroglyph, LLC, Kennewick, USA) coupled with a Q Exactive Plus orbitrap (Thermo Fisher Scientific Inc., Waltham, USA). Pierce Negative Ion Calibration Solution (Thermo Fisher Scientific Inc.) was used for external mass calibration. Raster step size was set to 75 µm. The mass range m/z 85–1000 was recorded with a fixed inject time of 250 ms and a mass resolution of 70,000 in negative ion mode.

### Data analysis

MALDI-Orbitrap-MSI data were converted to imzML format using the software Spectroglyph Image Insight Ver 0.1.0.17171 (Spectroglyph). For data exploration, TMAs were combined into one dataset using the software SCiLS Lab MVS 2020a Pro (Bruker Daltonik GmbH). A peak list was created manually to exclude artefacts and matrix peaks. Spectra were normalized to the total ion count and ion images were generated with a threshold of ±1 mDa. Analytes were putatively annotated by their accurate mass using METLIN [23] and the Human Metabolome Database (HMDB) [24].

Further data analysis was performed with Python 3.7 (Python Software Foundation, Wilmington, USA). MSI imzML data was imported using the pyimzML parser. A software solution was implemented for the automated co-registration of MSI data with digital pathology results from QuPath using OpenCV [25]. Spectral information was extracted for classified tumor regions of TMA cores for manually picked peaks (*n* = 199) and mean intensities for cores were calculated. Analytes with absolute mean intensities above 70 (*n* = 173) were used to calculate the feature importance by random forest classification with a threshold of 0.01, yielding 27 ion channels (Supplementary information). Different algorithms (k-nearest neighbors (KNN), support-vector machine (SVM), and random forest) were used to classify cases based on metabolic profiles using eightfold cross-validation. For this purpose, mean intensities for each case were calculated by combining core intensities. The diagnostic ability of the classifiers was visualized in a receiver operating characteristic (ROC) using scikit-learn [26]. Differences between groups were furthermore visualized via t-distributed stochastic neighbor embedding (t-SNE) [27] and boxplot analyses. Boxplots were generated with tumor cases, using mean intensities for each tumor. Statistical significance was calculated with the statannot package using Kruskal–Wallis test with Bonferroni correction.

## Results

In order to characterize UrC versus CRC, TMAs were established. First, thin sections were analyzed by the established histopathological classification upon H&E-staining. In a second step these TMAs were analyzed by MSI.

### Tissue specimen

Four TMAs (UrC: *n* = 2, CRC: *n* = 2), consisting of 146 TMA cores in total (UrC: *n* = 19 cases, CRC: *n* = 27 cases), were constructed for analyses. The cohort comprises 66 UrC cores, derived from 19 cases and 80 CRC cores from 27 tumor samples. All analyzed cores included tumor and non-tumor regions.

### Histopathological classification and transformation on mass spectrometry imaging data

**Fig. 1:**
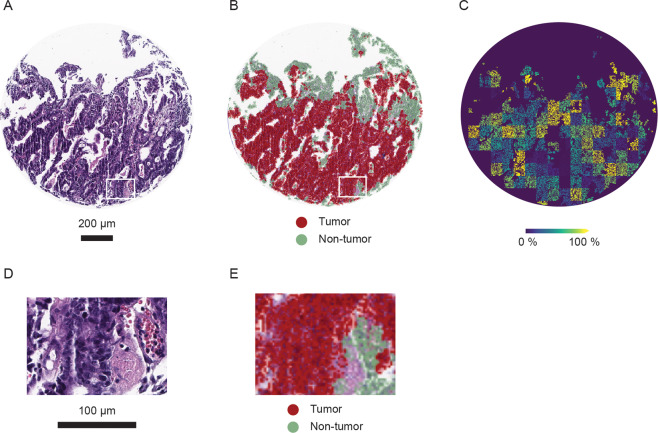
Results of histopathological classification and assignment on mass spectrometry results in an urachal adenocarcinoma (UrC) core. **A** Hematoxylin and eosin staining. Scale: 200 µm. **B** QuPath classification result. Red areas are classified as tumor and green areas as non-tumorous regions. **C** Mass spectrometry imaging result for m/z 214.0482 in classified tumor regions. **D** Area of zoom with higher magnification of hematoxylin and eosin staining. Corresponding area is annotated in Fig. 1A. Scale: 100 µm. **E** Area of zoom with higher magnification of the classification result. Corresponding area is annotated in Fig. 1B.

Cells from 146 TMA cores were automatically detected using the QuPath software and classified by utilizing cell features in a random trees algorithm on the basis of H&E images. In this way, stained tissue sections (Fig. 1A) were divided into tumor and non-tumor regions, e.g., stroma and necrotic tissue (Fig. 1B, D and E). The resulting mask of tumor regions was transferred onto MSI data after image transformation, minimizing the inclusion of non-tumor regions in data analysis (Fig. 1C).

### Differentiation of UrC and CRC through multivariate analyses

**Fig. 2:**
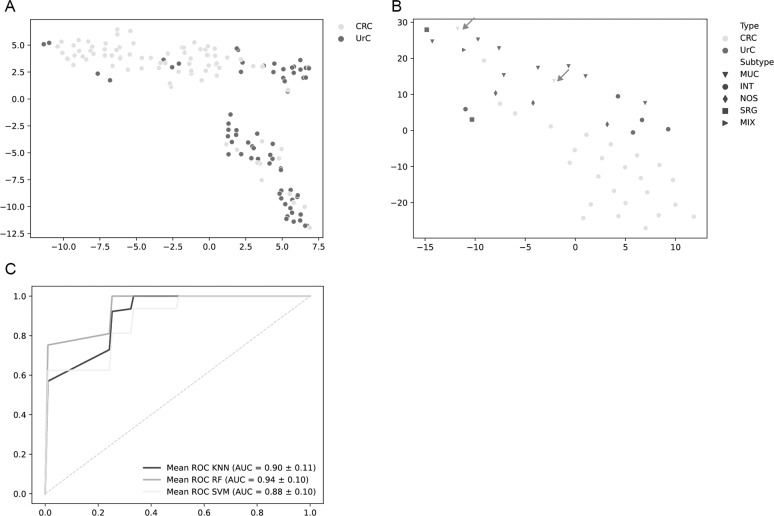
Visualization of metabolic differences between urachal adenocarcinomas (UrC) and colorectal adenocarcinomas (CRC). **A** t-distributed stochastic neighbor embedding (t-SNE) algorithm. Each dot represents one TMA core. UrC cores: *n* = 66, CRC cores: *n* = 80. **B** Visualization of cases via t-SNE including tumor subtypes. Arrows indicate mucinous CRC. UrC: *n* = 19, CRC: *n* = 27. **C** Receiver operating characteristic (ROC) analysis of a cross-validated k-nearest neighbors algorithm (black), random forest algorithm (dark gray), and support-vector machine algorithm (light gray) on tumor cases. UrC: *n* = 19, CRC: *n* = 27. MUC: mucinous subtype, INT: intestinal subtype, NOS: not otherwise specified subtype, SRG: signet-ring cell subtype, MIX: mixed subtype, KNN: k-nearest neighbors, RF: random forest, SVM: support-vector machine.

Metabolic differences in tumor regions from UrC and CRC tissues were demonstrated through multivariate analyses. Twenty-seven m/z channels were selected using feature importance and were used for the calculations (Supplementary information). Similarities between metabolic phenotypes are visualized via t-SNE algorithm. A separation of UrC and CRC cores can be recognized, however, transition between tumor groups shows overlaps (Fig. 2A). Considering tumor subtypes of all analyzed cases revealed that outliers particularly consist of mucinous CRC (*n* = 2), which seem to resemble metabolic profiles of mucinous UrC specimen (Fig. 2B).

Different classifiers were trained on the metabolite intensity data of UrC and CRC cases. Using cross-validation, a classification accuracy of 0.87 (±0.15) was yielded using a random forest algorithm, 0.87 (±0.22) using a KNN algorithm and 0.83 (±0.24) using a SVM algorithm. The corresponding ROC analysis describes the ability to distinguish between UrC and CRC tumors and shows an area under the curve (AUC) of 0.94 for the random forest classifier, 0.9 for the KNN classifier and 0.88 for the SVM classifier (Fig. 2C).

### UrC metabolite levels differ from CRC metabolite levels

**Fig. 3:**
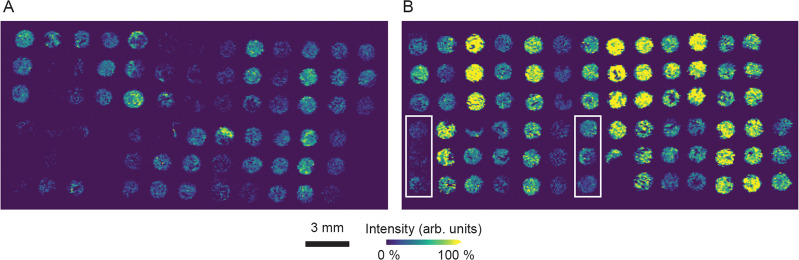
Ion image of m/z 124.0064, annotated as analyte taurine. **A** Urachal adenocarcinoma (UrC) TMA cores. **B** Colorectal adenocarcinoma (CRC) TMA cores. Mucinous CRC cases are highlighted in rectangles. Scale: 3 mm.

**Fig. 4:**
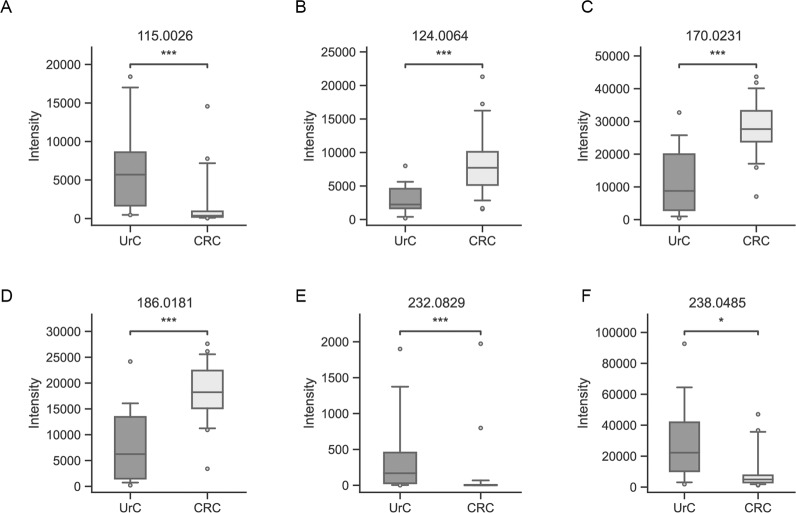
Boxplot analyses of metabolites, detected by MALDI-Orbitrap-MSI, extracted from classified tumor regions in urachal adenocarcinomas (UrC) and colorectal adenocarcinomas (CRC). Cores derived from one case were combined by mean intensities. **A** m/z 115.0026 (fumarate), (**B**) m/z 124.0064 (taurine), (**C**) m/z 170.0231 (adenine), (**D**) m/z 186.0181 (guanine), (**E**) m/z 232.0829, (**F**) 238.0485 (N-acetyl-L-2-aminoadipate). Statistical significance is indicated through asterisks with *p* < 0.05 as significant (Not significant: 0.05 ≤ *p* ≤ 1, *: 0.01 < *p* < 0.05, **: 0.001 < *p* ≤ 0.01, ***: 0.0001 < *p* ≤ 0.001, ****: *p* ≤ 0.0001). UrC: *n* = 19, CRC: *n* = 27.

Several metabolites were found to be significantly different in their abundance, when comparing UrC with CRC specimen. However, no analyte was found to be abundant uniquely in one tumor group. Antioxidant taurine (m/z 124.0064) shows higher signal intensities in cores of the CRC group (Fig. 3), which was verified through statistical analysis (*p* = 0.0009). A classification accuracy of 0.74 was achieved by a random forest classifier based solely on taurine levels. Intensity levels of taurine and further analytes that are significantly different in the tumors are visualized in boxplots (Fig. 4). Ion channels m/z 170.0231 and m/z 186.0188 are enhanced in CRC samples as well. These m/z values represent the chloride adduct ions of purine bases adenine and guanine with respective *p* values of 0.0003 and 0.0003 (Fig. 4C, D). Supporting these results, taurine, adenine, and guanine were detected as [M-H]^−^ and [M + Cl]^−^ ions, showing similar differences between groups. Therefore, only the ion channel with higher intensity is shown, respectively.

Furthermore, analytes were found to have higher abundances in UrC. The analyte with m/z 115.0026 was annotated as fumarate. The tricarboxylic acid cycle metabolite shows increased intensity levels in UrC specimen, compared to CRC specimen (*p* = 0.0006) (Fig. 4A). Likewise, ion channels m/z 232.0829 and m/z 238.0485, a N-acyl-alpha amino acid, show significantly higher levels in UrC specimen with *p* values of 0.0002 and 0.0341, respectively (Fig. 4E, F).

## Discussion

The histopathological differential diagnostic process of UrC is of major therapeutic importance. However, as supportive diagnostic technologies were shown to be helpful only in a subset of cases or specific settings [4, 28, 29], diagnostic biomarkers are urgently needed.

For the detection of diagnostic biomarkers in UrC, we therefore sought to employ a technology that has not yet been used in this setting (MALDI-MSI). Aims of the present study were to (i) show the feasibility of MALDI-MSI for the evaluation of FFPE tissue in UrC and CRC as its most relevant differential diagnosis, (ii) combine spatial MALDI-MSI data with annotated histopathological data from digitalized H&E slides, and finally (iii) evaluate metabolites as a differential diagnostic biomarker in UrC versus CRC.

Considering the first aim, the analysis of metabolites from FFPE tissue is still a great obstacle. Although the feasibility of MALDI-MSI for the evaluation of FFPE tissue was demonstrated in principle [22], it has to be noted that less metabolites are detectable via MS in FFPE samples compared to fresh frozen tissue [30]. However, in case of rare tumors such as UrC, where only few tissue samples are available over years, the use of FFPE material is inevitable. In this study, the less commonly used matrix NEDC [31] was utilized and a successful application on FFPE tissue samples was demonstrated. Considering these results, the present study is the first to use these techniques in this setting showing metabolic differences.

The metabolite with most prominent differential expression between UrC and CRC, taurine, is an amino acid with antioxidative properties, that can induce apoptosis and can suppress proliferation in tumor cells [32, 33]. Increased taurine levels in CRC in comparison to non-tumorous specimen were reported previously [34]. This emphasizes the need of spatially separating tumorous from non-tumorous tissue in the MALDI-MSI analysis to detect metabolites that derive from the cancer cells themselves but not from the stromal compartment or non-tumorous epithelia. We addressed this issue by H&E-staining of serial sections of the TMA used for MALDI-MSI analysis. These H&E slides were scanned, and cancer cells were digitally identified after training of an algorithm based on the pathologist’s annotations and manual validation of the final detection results. After merging the MALDI-MSI data with data from digital pathology, the metabolic profile could be spatially assigned to the cancer itself thus acting as a proof-of-concept of the study’s second aim to combine MALDI-MSI and histopathological data with spatial discrimination (Fig. 1). The relevance of this multimodal approach is emphasized, as the 2021a release of the commercially available MSI software SCiLS Lab (Bruker Daltonik GmbH) now also allows an export of QuPath annotations into SCiLS Lab.

Beside taurine, also several small molecules with significantly different levels in the two tumor types were detected. This is important for achieving the AUC of 0.94 in ROC-analyses (Fig. 2C). Best classification result was obtained using a random forest algorithm, which is limiting data-overfitting and was used in various MSI approaches before [35]. However, for taurine alone, the diagnostic accuracy was 74% with an AUC of 0.77 representing an excellent result in the present study setup. As the two tumor types also show an overlap in the t-SNE visualization, it is interesting to note that most outliers were of mucinous subtypes both in UrC and CRC with strong discrimination of intestinal type tumors (Fig. 2A and B). These differences have to be kept in mind when applying the technology in this scenario. However, considering the third aim of the study, the diagnostic accuracy of taurine levels measured by MALDI-MSI considerably outperforms currently available adjunctive technologies such as immunohistochemistry of beta-catenin or CK7 [4, 36, 37].

Taurine was additionally identified to be enhanced in urine samples of patients with a colorectal neoplasia [38]. In turn, the finding of lower taurine levels in UrC specimen might also be reflected in urinary samples and should be further analyzed.

Our study has some limitations. As stated above, data quality could be increased, if fresh frozen tissue of both UrC and CRC would have been used. However, as UrC is such a rare tumor type, FFPE tissue samples are the only available source of material in sufficient numbers. Although we additionally analyzed different cores per sample and discriminated tumorous from non-tumorous areas, the number of samples, i.e., cohort size, used in the present study still is low. Therefore, the results should be considered as a proof-of-concept with the result of a promising diagnostic biomarker (taurine) from a combined MALDI-MSI/digital pathology approach, which has to be validated in further studies and larger cohorts.

## Supplementary information

Supplementary Information

## Data Availability

The datasets used and analyzed in this study are available from the corresponding author on reasonable request.

## References

[1] Upadhyay V, Kukkady A (2003). Urachal remnants: an enigma. Eur J Pediatr Surg.

[2] Schubert GE, Pavkovic MB, Bethke-Bedürftig BA (1982). Tubular urachal remnants in adult bladders. J Urol.

[3] Szarvas T, Módos O, Niedworok C, Reis H, Szendröi A, Szász MA (2016). Clinical, prognostic, and therapeutic aspects of urachal carcinoma-A comprehensive review with meta-analysis of 1,010 cases. Urol Oncol.

[4] Reis H, Krafft U, Niedworok C, Módos O, Herold T, Behrendt M (2018). Biomarkers in Urachal Cancer and Adenocarcinomas in the Bladder: a Comprehensive Review Supplemented by Own Data. Dis Markers.

[5] Szarvas T, Reis H (2020). Editorial Comment from Dr Szarvas and Dr Reis to Clinicopathological features of malignant urachal tumor: A hospital-based cancer registry data in Japan. Int J Urol.

[6] Hager T, Kraywinkel K, Szarvas T, Hadaschik B, Schmid KW, Reis H (2020). Urachal Cancer in Germany and the USA: An RKI/SEER Population-Based Comparison Study. Urol Int.

[7] Paner GP, Lopez-Beltran A, Sirohi D, Amin MB (2016). Updates in the Pathologic Diagnosis and Classification of Epithelial Neoplasms of Urachal Origin. Adv Anat Pathol.

[8] Romero-Garcia S, Lopez-Gonzalez JS, Báez-Viveros JL, Aguilar-Cazares D, Prado-Garcia H (2011). Tumor cell metabolism: an integral view. Cancer Biol Ther.

[9] Hanahan D, Weinberg RA (2011). Hallmarks of cancer: the next generation. Cell.

[10] Yang L, Venneti S, Nagrath D (2017). Glutaminolysis: a Hallmark of Cancer Metabolism. Annu Rev Biomed Eng.

[11] Warburg O, Wind F, Negelein E (1927). The metabolism of tumors in the body. J Gen Physiol.

[12] Pavlova NN, Thompson CB (2016). The Emerging Hallmarks of Cancer Metabolism. Cell Metab.

[13] DeBerardinis RJ, Chandel NS (2016). Fundamentals of cancer metabolism. Sci Adv.

[14] Ren J-L, Zhang A-H, Kong L, Wang X-J (2018). Advances in mass spectrometry-based metabolomics for investigation of metabolites. RSC Adv.

[15] Schwamborn K. Chapter One—The Importance of Histology and Pathology in Mass Spectrometry Imaging. In: Drake RR & McDonnell, Liam A., editors. Advances in cancer research: applications of mass spectrometry imaging to cancer. Cambridge, Mass.; San Diego, Calif.; Oxford; London: Academic Press; 2017. p. 1–26. 10.1016/bs.acr.2016.11.001 28110647

[16] Ščupáková K, Balluff B, Tressler C, Adelaja T, Heeren RMA, Glunde K (2020). Cellular resolution in clinical MALDI mass spectrometry imaging: the latest advancements and current challenges. Clin Chem Labor Med.

[17] Jones MA, Cho SH, Patterson NH, van de Plas R, Spraggins JM, Boothby MR (2020). Discovering New Lipidomic Features Using Cell Type Specific Fluorophore Expression to Provide Spatial and Biological Specificity in a Multimodal Workflow with MALDI Imaging Mass Spectrometry. Analy Chem.

[18] Ščupáková K, Dewez F, Walch AK, Heeren RMA, Balluff B (2020). Morphometric Cell Classification for Single-Cell MALDI-Mass Spectrometry Imaging. Angew Chem Int Ed Engl.

[19] Moch H, Humphrey PA, Ulbright TM, Reuter VE. WHO classification of tumours of the urinary system and male genital organs. Lyon: IARC Press; 2016. 10.1016/j.eururo.2016.02.028 26996659

[20] Bosman FT. WHO classification of tumours of the digestive system. 4th ed. Lyon: IARC; 2010.

[21] Bankhead P, Loughrey MB, Fernández JA, Dombrowski Y, McArt DG, Dunne PD (2017). QuPath: open source software for digital pathology image analysis. Sci Rep.

[22] Buck A, Ly A, Balluff B, Sun N, Gorzolka K, Feuchtinger A (2015). High-resolution MALDI-FT-ICR MS imaging for the analysis of metabolites from formalin-fixed, paraffin-embedded clinical tissue samples. J Pathol.

[23] Smith CA, O’Maille G, Want EJ, Qin C, Trauger SA, Brandon TR (2005). METLIN: a metabolite mass spectral database. Ther Drug Monit.

[24] Wishart DS, Tzur D, Knox C, Eisner R, Guo AC, Young N (2007). HMDB: the Human Metabolome Database. Nucleic Acids Res.

[25] Bradski G. The OpenCV Library. Dr. Dobb’s Journal of Software Tools 2000. http://citebay.com/how-to-cite/opencv/.

[26] Pedregosa F, Varoquaux G, Gramfort A, Michel V, Thirion B, Grisel O (2011). Scikit-learn: machine Learning in Python. J Mach Learn Res.

[27] van der Maaten L, Hinton G (2008). Visualizing Data using t-SNE. J Mach Learn..

[28] Maurer A, Ortiz-Bruechle N, Guricova K, Rose M, Morsch R, Garczyk S (2020). Comparative genomic profiling of glandular bladder tumours. Virchows Arch.

[29] Thiem S, Herold T, Krafft U, Bremmer F, Tolkach Y, Szász AM (2017). Telomerase reverse transcriptase (TERT) promoter mutations are rare in urachal cancer. Pathol Int.

[30] Cacciatore S, Zadra G, Bango C, Penney KL, Tyekucheva S, Yanes O (2017). Metabolic Profiling in Formalin-Fixed and Paraffin-Embedded Prostate Cancer Tissues. Mol Cancer Res.

[31] Wang J, Qiu S, Chen S, Xiong C, Liu H, Wang J (2015). MALDI-TOF MS imaging of metabolites with a N-(1-naphthyl) ethylenediamine dihydrochloride matrix and its application to colorectal cancer liver metastasis. Analyt Chem.

[32] Tu S, Zhang X-L, Wan H-F, Xia Y-Q, Liu Z-Q, Yang X-H (2018). Effect of taurine on cell proliferation and apoptosis human lung cancer A549 cells. Oncol Lett.

[33] Zhang X, Tu S, Wang Y, Xu B, Wan F (2014). Mechanism of taurine-induced apoptosis in human colon cancer cells. Acta Biochimica et Biophysica Sinica.

[34] Righi V, Durante C, Cocchi M, Calabrese C, Di Febo G, Lecce F (2009). Discrimination of healthy and neoplastic human colon tissues by ex vivo HR-MAS NMR spectroscopy and chemometric analyses. J Proteome Res.

[35] Zhang Y, Liu X (2018). Machine learning techniques for mass spectrometry imaging data analysis and applications. Bioanalysis.

[36] Reis H, Szarvas T (2019). Das Urachuskarzinom – aktuelle Konzepte einer seltenen Tumorerkrankung. Pathologe.

[37] Nagy N, Reis H, Hadaschik B, Niedworok C, Módos O, Szendrői A (2020). Prevalence of APC and PTEN Alterations in Urachal Cancer. Pathol Oncol Res.

[38] Kim ER, Kwon HN, Nam H, Kim JJ, Park S, Kim Y-H (2019). Urine-NMR metabolomics for screening of advanced colorectal adenoma and early stage colorectal cancer. Sci Rep.

[39] Sheldon CA, Clayman RV, Gonzalez R, Williams RD, Fraley EE (1984). Malignant urachal lesions. J. Urol.

[40] Ashley RA, Inman BA, Sebo TJ, Leibovich BC, Blute ML, Kwon ED (2006). Urachal carcinoma: clinicopathologic features and long-term outcomes of an aggressive malignancy. Cancer.

[41] Brierley J, Gospodarowicz MK, Wittekind C. TNM classification of malignant tumours. In: Brierley J, Gospodarowicz MK, Wittekind C, editors. Chichester, West Sussex, UK, Hoboken, NJ: John Wiley & Sons Inc, 2017.

