# Correction to: A new technological approach in diagnostic pathology: mass spectrometry imaging-based metabolomics for biomarker detection in urachal cancer

**DOI:** 10.1038/s41374-021-00625-2

**Published:** 2021-06-21

**Authors:** Judith Martha Neumann, Karsten Niehaus, Nils Neumann, Hans Christoph Knobloch, Felix Bremmer, Ulrich Krafft, Udo Kellner, Peter Nyirády, Tibor Szarvas, Hanna Bednarz, Henning Reis

**Affiliations:** 1 Proteome and Metabolome Research, Center for Biotechnology (CeBiTec), Faculty of Biology, Bielefeld University, Bielefeld, Germany; 2 Research Institute for Cognition and Robotics (CoR-Lab), Bielefeld University, Bielefeld, Germany; 3 Department of Urology, University Medical Center Göttingen, University of Göttingen, Göttingen, Germany; 4 Institute of Pathology, University Medical Center Göttingen, University of Göttingen, Göttingen, Germany; 5 Department of Urology, University Hospital Essen, University of Duisburg-Essen, Essen, Germany; 6 Institut für Pathologie, Johannes Wesling Klinikum Minden, Minden, Germany; 7 Department of Urology, Semmelweis University Budapest, Budapest, Hungary; 8 Medical School OWL, Bielefeld University, Bielefeld, Germany; 9 Institute of Pathology, University Hospital Essen, University of Duisburg-Essen, Essen, Germany

**Keywords:** Cancer metabolism, Tumour biomarkers, Urological cancer

Correction to: *Laboratory Investigation* 10.1038/s41374-021-00612-7, published online 21 May 2021

The original version of this article unfortunately contained a mistake. Due to the submission of Table 1 in a separate file, the following citations were missing in the references, leading to an incorrect linking in the table caption. The missing reference are:

(A) Sheldon CA, Clayman RV, Gonzalez R, Williams RD, Fraley EE. Malignant urachal lesions. J Urol. 131, 1–8 (1984).

(B) Ashley RA, Inman BA, Sebo TJ, Leibovich BC, Blute ML, Kwon ED, et al. Urachal carcinoma: clinicopathologic features and long-term outcomes of an aggressive malignancy. Cancer. 107, 712–720 (2006).

(C) Brierley J, Gospodarowicz MK, Wittekind C. TNM classification of malignant tumors. In: Brierley J, Gospodarowicz MK, Wittekind C, editors. Chichester, West Sussex, UK, Hoboken, NJ: John Wiley & Sons Inc; 2017.

The authors apologize for the mistake. The original article has been corrected.

