# Supplementary material for: A new technological approach in diagnostic pathology: mass spectrometry imaging-based metabolomics for biomarker detection in urachal cancer
Source: Lab Invest. 2021 May 21;101(9):1281–8. doi: 10.1038/s41374-021-00612-7 (PMC8367814; doi:10.1038/s41374-021-00612-7)
Supplement: Supplementary file 1 — Supplementary Information [file 41374_2021_612_MOESM1_ESM.pdf]

Supplementary information

Measured m/z	Calculated m/z	Sum formula	Analyte	Adduct	Mass error (ppm)
115.0026	115.0037	$C_4H_4O_4$	Fumarate	$[M-H]^-$	-9.4
124.0064	124.0074	$C_2H_7NO_3S$	Taurine	$[M-H]^-$	-7.7
134.0461	134.0472	$C_5H_5N_5$	Adenine	$[M-H]^-$	-8.2
136.0061	-	-	-	-	-
138.0221	-	-	-	-	-
146.0450	146.0459	$C_5H_9NO_4$	Glutamate	$[M-H]^-$	-5.9
150.0413	150.0421	$C_5H_5N_5O$	Guanine	$[M-H]^-$	-5.3
159.9831	159.9841	$C_2H_7NO_3S$	Taurine	$[M+Cl]^-$	-6.3
161.9801	-	-	-	-	-
170.0231	170.0239	$C_5H_5N_5$	Adenine	$[M+Cl]^-$	-4.7
172.0201	-	-	-	-	-
175.9881	-	-	-	-	-
186.0181	186.0188	$C_5H_5N_5O$	Guanine	$[M+Cl]^-$	-3.8
192.0662	-	-	-	-	-
193.0352	193.0354	$C_6H_{10}O_7$	-	$[M-H]^-$	-1
195.0506	195.0510	$C_6H_{12}O_7$		$[M-H]^-$	-2.1
197.0032	-	-	-	-	-
207.9972	-	-	-	-	-
212.9722	-	-	-	-	-
214.9692	-	-	-	-	-
221.9952	-	-	-	-	-
232.0829	-	-	-	-	-

238.0485	238.0488	$C_8H_{13}NO_5$	N-acetyl-L-2-aminoadipate	$[M+Cl]^-$	-1.1
263.0963	-	-	-	-	-
282.0753	-	-	-	-	-
419.2569	419.2569	C21H41O6P	Cyclic Phosphatidic acid (18:0)	$[M-H]^-$	0
885.5495	885.5498	$C_{47}H_{83}O_{13}P$	Phosphatidylinositol (38:4)	$[M-H]^-$	-0.3
